# Analysis of Membrane Type-1 Matrix Metalloproteinase (MT1-MMP, MMP14) in Eutopic and Ectopic Endometrium and in Serum and Endocervical Mucus of Endometriosis

**DOI:** 10.3390/biomedicines11102730

**Published:** 2023-10-09

**Authors:** Jane B. Maoga, Muhammad A. Riaz, Agnes N. Mwaura, Ezekiel Mecha, Charles O. A. Omwandho, Georgios Scheiner-Bobis, Ivo Meinhold-Heerlein, Lutz Konrad

**Affiliations:** 1 Center of Gynecology and Obstetrics, Faculty of Medicine, Justus Liebig University Giessen, 35392 Giessen, Germany; jane.maoga@childrens.harvard.edu (J.B.M.); muhammad.a.riaz@gyn.med.uni-giessen.de (M.A.R.); agnes.mwaura@ubc.ca (A.N.M.); ivo.meinhold-heerlein@gyn.med.uni-giessen.de (I.M.-H.); 2 Department of Biochemistry, University of Nairobi, Nairobi P.O. Box 30197-00100, Kenya; emecha@uonbi.ac.ke; 3 Department of Health Sciences, Kirinyaga University, Kerugoya P.O. Box 143-10300, Kenya; omwandho@kyu.ac.ke; 4 Institute for Veterinary Physiology and Biochemistry, School of Veterinary Medicine, Justus Liebig University Giessen, 35392 Giessen, Germany; georgios.scheiner-bobis@vetmed.uni-giessen.de

**Keywords:** endometrium, endometriosis, adenomyosis, MT1-MMP

## Abstract

Background: Membrane type-matrix metalloproteinases (MT-MMPs) are a subgroup of the matrix metalloproteinases (MMPs) family and are key molecules in the degradation of the extracellular matrix. Membrane type-1 matrix metalloproteinase (MT1-MMP, MMP14) is often deregulated in different cancer tissues and body fluids of human cancer patients; however, MT1-MMP levels in endometriosis and adenomyosis patients are currently unknown. Materials and Methods: Tissue samples from patients with and without endometriosis or adenomyosis were analyzed with immunohistochemistry for the localization of MT1-MMP. Serum and endocervical mucus samples from patients with and without endometriosis or adenomyosis were investigated with MT1-MMP ELISAs. Results: MT1-MMP was localized preferentially in the glands of eutopic and ectopic endometrium. MT1-MMP protein levels are significantly reduced in ovarian endometriosis (HSCORE = 31) versus eutopic endometrium (HSCORE = 91) and adenomyosis (HSCORE = 149), but significantly increased in adenomyosis (HSCORE = 149) compared to eutopic endometrium (HSCORE = 91). Similarly, analysis of the levels of MT1-MMP using enzyme-linked immune assays (ELISAs) demonstrated a significant increase in the concentrations of MT1-MMP in the serum of endometriosis patients (1.3 ± 0.8) versus controls (0.7 ± 0.2), but not in the endocervical mucus. Furthermore, MT1-MMP levels in the endocervical mucus of patients with endometriosis were notably reduced in patients using contraception (3.2 ± 0.4) versus those without contraception (3.8 ± 0.2). Conclusions: Taken together, our findings showed an opposite regulation of MT1-MMP in the tissue of ovarian endometriosis and adenomyosis compared to eutopic endometrium without endometriosis but increased serum levels in patients with endometriosis.

## 1. Introduction

Endometriosis is a benign condition in which endometrial-like tissue is located outside the uterus, usually in the pelvis, but occasionally also in the lungs, liver, colon, and brain [1,2,3]. A recent review found that endometriosis affects 0.7–8.6% of women of childbearing age [4]. Chronic pelvic pain, dyspareunia, dysmenorrhea, dyschezia, and infertility are the most commonly reported symptoms among patients [3,4]. There are several theories regarding the pathogenesis of endometriosis, with retrograde menstruation with implantation of endometrial tissue outside the uterus being the most widely accepted theory [5].

Adenomyosis, also known as endometriosis interna, is characterized by an enlarged uterus with hyperplastic and hypertrophic myometrium resulting from the occurrence of endometrial tissue in the myometrium. It frequently co-occurs with other gynecological conditions and is associated with unusual uterine bleeding, persistent pelvic pain, and infertility [6,7,8,9]. The incidence rates in hysterectomy are 20–30% [10]. There are two main theories for the development of adenomyosis: invagination and metaplasia [7,9,11]. However, recent 3D reconstructions of the uterus suggest invagination is the most important, if not the only cause [12].

In the human endometrium, matrix metalloproteinases (MMPs) are secreted almost exclusively by stromal cells, with the exception of MMP7, which is synthesized by endometrial epithelial cells [13]. The involvement of MMPs in endometrial remodeling, menstruation, and endometriosis has been described [14,15], although it is still unclear which MMPs are responsible for tissue breakdown during menstruation [16]. MMPs are a group of enzymes mainly involved in extracellular matrix (ECM) remodeling but also promote cell migration, proliferation, invasion, angiogenesis, and differentiation [17]. Recently, a higher mRNA expression of MMP2 and MMP9 was detected in menstrual blood-derived stromal cells from women with endometriosis compared to patients without endometriosis [18].

MT-MMPs represent a subclass of the MMPs, which is further subdivided into the transmembrane- and glycosylphosphatidylinositol (GPI)-anchored MT-MMPs [19]. Due to their membrane localization, they perform different roles, including cleavage and activation of different cytokines, receptors, and growth factors on the cell surface [17,20]. Tissue inhibitors of matrix metalloproteinases (TIMPs) maintain an equilibrium between TIMPs and MMPs expression by binding to them in a 1:1 ratio. Alterations of this balance are associated with different pathological conditions [17,21].

Six MT-MMPs have been described in humans, and MT1-MMP is the most widely studied [19]. It degrades different components of the fibrillary collagens, such as type I-III collagen, but not type IV collagen. However, activated MMP2 can degrade type IV collagen [19]. MT1-MMP null mice show impaired ossification and alveolization and alterations in the cytoskeleton and lamina structure [22,23,24]. Furthermore, impaired MT1-MMP expression has been linked to the pathogenesis of various health conditions, including cancer and obesity [25,26].

In the human endometrium, MT1-MMP expression in different cellular compartments, such as leukocytes, epithelial, luminal, and stromal cells, has been reported [27,28,29]. MT1-MMP mRNA was expressed throughout the cycle, and the protein was found in epithelial and stromal cells [27]. Interestingly, MT1-MMP mRNA/protein expression is upregulated in ectopic endometrium and pigmented endometriotic lesions compared to eutopic endometrium, as well as in cases with endometriosis compared to those without endometriosis [29,30,31]. On the other hand, low levels of MT1-MMP and MMP13 in the peritoneal fluid of patients with endometriosis versus those without endometriosis have been reported [32].

Although MT1-MMP protein and mRNA expression has been described in endometriosis, their expression in adenomyosis has not been explored. Similarly, the concentrations of MT1-MMP in the serum and endocervical mucus in endometriosis and whether MT1-MMP can be used for a diagnostic non-invasive test has not been investigated. Consequently, in this retrospective study, we investigated the expression and localization pattern of MT1-MMP in endometriosis and adenomyosis. The levels of MT1-MMP in serum and endocervical mucus samples were also analyzed.

## 2. Materials and Methods

### 2.1. Patients and Sample Collection

The current study was approved by the ethics committee of the medical faculty of Justus Liebig University, Giessen, Germany (95/09). The study started in September 2009 and is still ongoing; however, not all patients could be included because tissue samples are preferentially preserved for the pathologist. In contrast to fixation with formalin, we prefer fixation with Bouin, which yields superior immunohistochemistry results [33]. All patients involved in the study gave written informed consent. Tissue samples (Table 1) were obtained from patients undergoing laparoscopy or hysterectomy because of pelvic-pain-related symptoms. We analyzed samples from the eutopic endometrium (EM) of patients with (EM EN^+^) and without (EM EN^−^) endometriosis, ovarian endometriosis (OV), and adenomyosis (AM) (Table 1). Eutopic endometrial samples of patients who had endometriosis and endometrial samples of patients who had adenomyosis were grouped together. Endometriosis was diagnosed by histological evaluation after laparoscopy, whereas the phase (proliferative and secretory) of the endometrial tissue was based on the dates of the last period and histological evaluation by the pathologist. Adenomyosis was diagnosed by histological evaluation by the pathologist after a hysterectomy. The intraoperative findings were classified according to the revised American Society for Reproductive Medicine score (rASRM) and ENZIAN score [34]. We used the following inclusion criteria: all premenopausal women at a fertile age with pelvic pain (mainly dysmenorrhea) and infertility problems, and all women who have been transferred to our endometriosis center due to abdominal problems by established doctors. None of the women used any hormone medication three months before surgery. We used the following exclusion criteria: patients suffering from cancer, pregnant women, women with a pelvic laparoscopy within 6 months of visiting our center, women with bladder infections, women suffering from nutcracker syndrome, and women with menopause. We also collected blood and endocervical mucus samples from healthy volunteers.

The specimens were fixed in Bouin’s solution and embedded in paraffin. Then, histological evaluation was performed following staining of 5 µm sections with hematoxylin and eosin.

Endocervical mucus (n = 193) and serum (n = 132) samples were obtained from patients during their clinical examination (Table 2). Patients on hormonal treatment included those on different contraceptives such as ethnylestradiol, dienogest, and progesterone-based contraceptives. Serum and endocervical mucus samples were obtained from patients as previously described [35]. MT1-MMP levels in the serum and endocervical mucus were determined using the human total MT1-MMP/MMP14 DuoSet ELISA (DY918-05, R&D Systems, Nordenstadt, Germany) following the manufacturer’s guidelines.

### 2.2. Immunohistochemical Analysis and Quantification

The immunohistochemistry of Bouin-fixed specimens was performed as previously described [33]. The EnVision Plus system (cat-no K4002, DAKO, Hamburg, Germany) together with diaminobenzidine (liquid DAB K3468, DAKO) were used according to the manufacturer’s instructions. The primary antibody against MT1-MMP (diluted 1:50, cat no PAB18771, Abnova, Taipeh, Taiwan) was used, but it was omitted in the negative control. Counterstaining of the tissue samples was performed using Meyer’s hematoxylin. Digital images were obtained using Leica DM 2000/Leica MC170/Leica application suite LAS 4.9.0 (Leica, Wetzlar, Germany) and processed with Adobe photoshop CS6. Quantification of MT1-MMP was performed using the percentage of stained glands and HSCORE (no staining = 0, weak but detectable = 1, moderate = 2, and intense = 3), which was calculated by adding up the percentage of cells in each category multiplied by the intensity of staining, giving a value between 0 and 300. All glands and cysts were included in the calculation of the HSCORE and percentage of stained glands.

### 2.3. MT1-MMP ELISA

Levels of MT1-MMP in the serum and endocervical mucus samples were quantified using the human total MMP14/MT1-MMP Duoset ELISA (DY918-05, range 0.625–20 ng/mL, R&D Systems, Nordenstadt, Germany) according to the manufacturer’s instructions. Absorbance was determined using the M200 microplate reader (Tecan, Männedorf, Switzerland) set at 450 nm/540 nm.

### 2.4. Statistics

GraphPad Prism software (version 5.01 Inc., La Jolla, CA, USA) was used for statistical analysis. The comparison of the mean values between different groups was performed using the Mann–Whitney and/Kruskal–Wallis tests. Spearman’s correlation test was used to analyze the correlation of MT1-MMP levels in the serum and endocervical mucus samples with cycle days. The *p*-values ≤ 0.05 were considered statistically significant. The sample size was calculated with the following formula: samples size = [z^2^ SD(1-SD)]/ME^2^ (z = 1.96 for a confidence interval of 95%; SD = standard deviation of 0.5, and EM = error margin of 0.1), as given in www.qualtrics.com (accessed on 25 October 2022). A sample size of n = 96 would have been sufficient for a confidence interval of 95%, a 50% standard deviation, and a 10% error margin. We used a sample size of n = 132 for the serum samples and n = 193 for the mucus samples.

## 3. Results

### 3.1. Identification of MT1-MMP Localization in the Human Uterus and Ovarian Endometriosis

**Figure 1:**
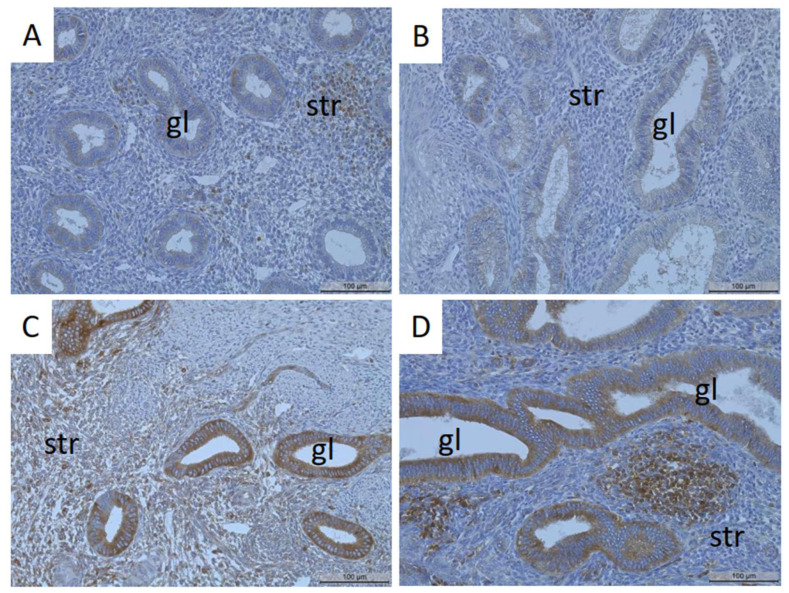

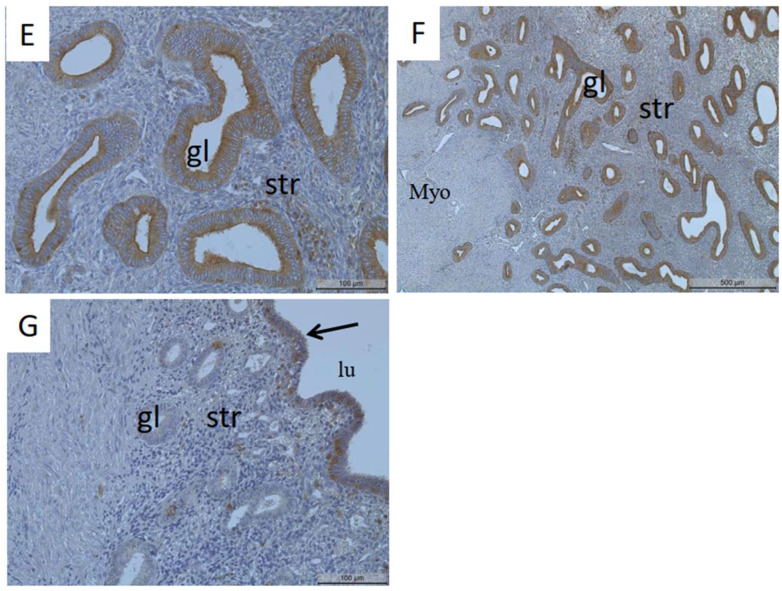
Faint staining of MT1-MMP in the glands of the eutopic endometrium of patients without endometriosis and without adenomyosis during the proliferative (**A**) and secretory (**B**) phases. Strong to moderate detection of MT1-MMP in the glands of the proliferative (**C**) and secretory (**D**) endometrium of patients with endometriosis but without adenomyosis. Some stromal cells showed strong to moderate MT1-MMP staining (**A**,**C**–**F**). Endometrial glands of patients with adenomyosis and with endometriosis showed strong MT1-MMP staining (**E**,**F**). Some luminal epithelial cells were also stained ((**G**) arrow, endometrium without endometriosis). Gl, gland; str, stroma; lu, lumen; myo, myometrium. Magnification: 20× (**A**–**E**,**G**) and 50× (**F**). Scale bars: 100 µm (**A**–**E**,**G**) and 500 µm (**F**).

MT1-MMP protein was mainly localized in the glandular epithelial cells of the proliferative and secretory endometrium and partly in the stromal and luminal epithelial cells (Figure 1A–G). Analysis of MT1-MMP localization in cases without endometriosis showed faint or no staining of the glands in the proliferative and secretory endometrium (Figure 1A,B). In cases with endometriosis, strong MT1-MMP staining was detected in the glandular epithelial cells of the proliferative and secretory endometrium (Figure 1C,D). Notably, glandular epithelial cells of the endometrium of patients with adenomyosis also demonstrated strong MT1-MMP staining (Figure 1E,F). Some staining was also observed in some stromal (Figure 1A,C–F) and luminal epithelial cells (Figure 1G).

**Figure 2:**
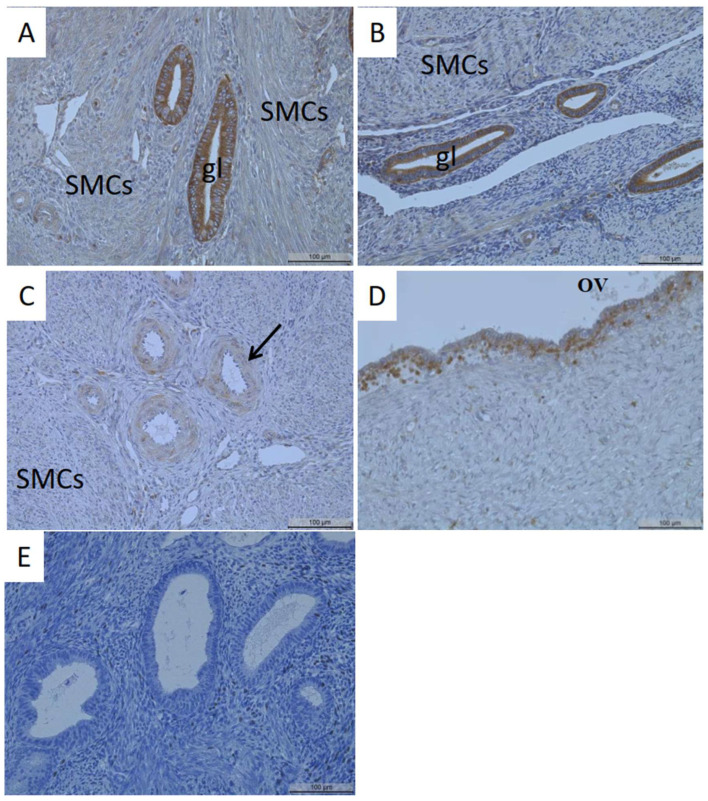
Strong MT1-MMP staining of the adenomyotic glands in the proliferative (**A**) and secretory (**B**) phases of patients with adenomyosis. The smooth muscle cells (**A**–**C**) and the blood vessels showed faint or no staining ((**C**), arrow). Strong to moderate detection of MT1-MMP in the epithelial cells of a cyst from a patient with ovarian endometriosis (**D**). A representative photo of the negative control (**E**). Gl, gland; SMCs, smooth muscle cells; OV, ovarian endometriosis. Magnification: 20×. Scale bars: 100 µm.

Comparable to patients with endometriosis, the glands of patients with adenomyotic lesions were also strongly stained (Figure 2A,B). The smooth muscle cells of the myometrium (Figure 2A–C) and the blood vessels (Figure 2C) showed faint/no MT1-MMP staining. MT1-MMP localization in ovarian endometriosis demonstrated MT1-MMP positivity in epithelial and some stromal cells (Figure 2D).

We combined datasets of eutopic endometrium samples (with and without endometriosis) and compared them with adenomyosis as well as ovarian endometriosis. The quantification of MT1-MMP staining showed a significantly higher HSCORE and percentage of stained glands in adenomyosis compared to ovarian endometriosis and eutopic endometrium (Table 3). Furthermore, the MT1-MMP HSCORE was significantly reduced in ovarian endometriosis versus eutopic endometrium (Table 3). The percentage of MT1-MMP-stained glands was also remarkably lower in ovarian endometriosis versus adenomyosis and higher in adenomyosis compared to the eutopic endometrium (Table 3).

### 3.2. Quantification of MT1-MMP in Serum and Endocervical Mucus Samples of Patients with and without Endometriosis

Especially in the proliferative phase, women with endometriosis showed higher MT1-MMP serum levels compared to women without endometriosis (Table 4). The mean MT1-MMP levels were higher in patients with endometriosis versus those without endometriosis (Table 5A). Women with endometriosis using contraception experienced significantly higher serum levels compared to women without endometriosis and without contraception (Table 5B).

A total of 193 endocervical mucus samples were analyzed for MT1-MMP levels (Table 2). Comparison of MT1-MMP levels in the cycle phases showed a rise in MT1-MMP levels of 46% and 31% during the proliferative phase of patients without and with endometriosis, respectively, as compared to the secretory phase (Table 6).

MT1-MMP levels in the endocervical mucus of patients with and without endometriosis were similar (Table 7A). However, MT1-MMP levels in the endocervical mucus of patients with endometriosis were 16% lower in cases using contraception compared to those without contraception (Table 7B).

We did not detect any differences in the MT1-MMP levels in the serum and endocervical mucus of patients with and without endometriosis with respect to BMI or age (Table 8A,B). There was also no correlation between the MT1-MMP levels in the serum or endocervical mucus of patients with respect to pain (dysmenorrhea, dysuria, dyschezia, and dyspareunia, Table 8A,B).

## 4. Discussion

In the present study, we investigated the expression and localization pattern of MT1-MMP in the eutopic endometrium of patients with and without endometriosis, adenomyosis, and ovarian endometriosis. Similarly, we analyzed the concentrations of MT1-MMP in the serum and endocervical mucus samples of patients with and without endometriosis.

### 4.1. Localization of MT1-MMP in the Uterus

Our results demonstrate that MT1-MMP protein is localized in the human endometrium across the different phases of the menstrual cycle, in agreement with previous reports [27,28,29,30,31,36]. Comparable to the research by Plaisier et al. [28] and Zhang et al. [27], MT1-MMP was preferentially localized in endometrial epithelial cells compared to stromal cells. Previous studies have reported that MT1-MMP protein was nearly absent in the endothelial cells of the blood vessel walls and smooth muscle cells of the myometrium [27,28], consistent with our results. However, Plaisier et al. [28] showed the presence of MT1-MMP protein in the perivascular smooth muscle cells of the myometrium. Although we did not compare stromal MT1-MMP staining intensity between patients with and without endometriosis, MT1-MMP stromal staining in cases without endometriosis was relatively faint/weak.

### 4.2. Localization of MT1-MMP in Adenomyosis and Endometriosis

MT1-MMP is involved in epithelial–mesenchymal transition, cell migration, invasion, and proliferation [37,38], and its upregulation in cancer is often correlated with poor prognosis [39,40,41]. Thus, we have analyzed MT1-MMP in adenomyosis and endometriosis. We report for the first time MT1-MMP protein localization in epithelial cells of adenomyotic lesions. Of note was the increased MT1-MMP expression in adenomyosis versus the eutopic endometrium of patients with and without endometriosis. Matsuda et al. [42] found increased MT1-MMP mRNA expression in the uteri of mice with induced adenomyosis compared to control uteri. MT1-MMP is involved in the activation of proMMP2 [19], and it is often linked to increased activation and expression of MMP2 in cases with endometriosis versus controls [30,31]. We suggest that the increased MT1-MMP expression in adenomyosis observed in our study could be responsible for activation and increased MMP2 expression in adenomyosis [43,44]. In endometriosis, past studies have shown upregulation of MT1-MMP protein/mRNA in patients with endometriosis compared to controls [30,31,32]. In contrast to the findings of Londero et al. [29], we observed reduced MT1-MMP expression in ovarian endometriosis as compared to eutopic endometrium and adenomyosis. However, Londero et al. [29] did not explore MT1-MMP in adenomyosis. Our present findings support our recent hypothesis [45,46] and that of Chung et al. [30] that most changes in expression patterns happen after implantation and are due to different microenvironments [47].

### 4.3. Serum Levels of MT1-MMP in Cases with Endometriosis

In the current study, MT1-MMP levels in the serum were higher in the menstrual phase versus the secretory phase only. Similarly, MT1-MMP mRNA expression is higher during the menstrual phase versus the secretory and proliferative phases [36].

Our results show significantly higher MT1-MMP levels in the serum samples of patients with endometriosis versus those without endometriosis, consistent with previous studies on cancer and preeclampsia patients [48,49,50,51,52]. However, in our study, one patient with endometriosis had considerably higher MT1-MMP levels (57.2 ng/mL) in the serum compared to the other patients, and we could not find any specific or special clinical characteristic of the patient in our database that could explain such high levels. In endometriosis, MT1-MMP and MMP13 concentrations are lower in the peritoneal fluid of patients with endometriosis compared to healthy controls [32]. 

### 4.4. Endocervical Mucus Samples of MT1-MMP in Endometriosis

In our study, endocervical mucus MT1-MMP levels were increased during the proliferative phase compared to the secretory phase and were highly similar in patients with and without endometriosis. Interestingly, we observed significantly reduced MT1-MMP levels in the endocervical mucus of patients with endometriosis using contraception versus those who were not using contraception. In the past, we have shown that contraceptives, especially dienogest alone or together with ethinylestradiol, reduced clusterin levels in the endocervical mucus of cases with endometriosis [53]. On the other hand, administration of different concentrations of estradiol valerate/dienogest oral contraceptives reduced cervical mucus production [54]. Therefore, we propose that the decreased levels of MT1-MMP in cases with endometriosis using contraception could be caused by contraceptives, possibly by reducing the thickness of the endometrium [54], resulting in reduced clusterin levels [53]. However, this has to be confirmed in further experiments, such as in primary and immortalized human endometrial cells. Moreover, MMPs and TIMPs in the cervical mucus plug have been associated with cervical remodeling and proteolytic processing in pregnancy and preterm labor [55].

### 4.5. Correlation of Serum/Endocervical Mucus Levels of MT1-MMP with Clinical Parameters and Clinical Implications

There were no associations between serum or endocervical mucus MT1-MMP levels and cycle day, BMI, or pain (dysuria, dysmenorrhea, dyschezia, and dyspareunia) in agreement with earlier findings in gastric cancer patients [49]. In uterine leiyomyoma, MT1-MMP mRNA expression is directly correlated with that of myostatin and activin A and intense dysmenorrhea [56].

The importance of MT1-MMP in tumor metastasis has increased significantly in recent years [57]. Not only the degradation of collagen type 1–3 is important, but also the involvement of the enzyme in the migration and invasion of tumor cells has raised hopes for a successful therapy with specific inhibitors [57]. Thus, our observation of an increased abundance of MT1-MMP, especially in adenomyosis, might be interesting for the development of future therapies against the disease.

### 4.6. Strengths and Limitations

In our observational retrospective study, we were the first to compare the tissue abundance of MT1-MMP in adenomyosis and compare it to eutopic endometrium and ectopic endometrium; however, due to the scarcity of material, it was not possible to analyze a larger number of individuals to reach the required number of patients. In the case of serum and endocervical mucus samples, it was possible to recruit a sufficiently high number of patients and healthy volunteers. Although we found some differences in values between patients with and without endometriosis, they are not usable for a noninvasive diagnostic test.

## 5. Conclusions

Our findings show localization of MT1-MMP mainly in epithelial cells of eutopic and ectopic endometrium. Moreover, we detected MT1-MMP in the serum and endocervical mucus of patients with endometriosis as well as those without endometriosis. This study was limited to in vivo studies in tissue, serum, and endocervical mucus samples; hence, further studies on the possible function of MT1-MMP in endometriosis and adenomyosis, especially in isolated endometrial/endometriotic cells as well as animal models, are necessary.

## Figures and Tables

**Table 1:** Summary of the tissue samples used for MT1-MMP.

Tissues	EM/EN^−^/AM^−^	EM/EN^−^/AM^+^	EM EN^+^/AM^−^	EM EN^+^/AM^+^	OV
All samples	n = 3	n = 12	n = 5	n = 8	n = 6
Median age ± SD	45 ± 4.4	41.5 ± 4.7	43 ± 2.8	46.5 ± 3.8	38 ± 8.4
Proliferative	n = 2	n = 7	n = 2	n = 6	
Secretory	n = 1	n = 2	n = 2	n = 1	
Unknown		n = 3	n = 1	n = 1	

EM EN^−^/AM^−^, endometrium without endometriosis and without adenomyosis; EM EN^−^/AM^+^, endometrium without endometriosis but with adenomyosis; EM EN^+^/AM^−^, endometrium with endometriosis but without adenomyosis; EM EN^+^/AM^+^, endometrium with endometriosis and with adenomyosis; OV, ovarian endometriosis; SD, standard deviation; n, number of samples.

**Table 2:** Overview of the serum and endocervical mucus samples used for MT1-MMP ELISAs.

	Serum Samples		Mucus Samples	
	EN^−^	EN^+^	EN^−^	EN^+^
(n)	61	71	87	106
Median age ± SD	27 ± 7.9	34 ± 7.1	28 ± 8.2	33 ± 7.1
BMI (kg/m^2^)	21.8 ± 6.3	23.7 ± 5.2	21.8 ± 4.6	22.9 ± 4.4
Smoking n (%)	n = 12 (19.7)	n = 17 (23.9)	n = 23 (26.4)	n = 24 (22.6)
Allergy n (%)	n = 31 (50.8)	n = 34 (47.9)	n = 44 (50.6)	n = 58 (54.7)
Menstrual phase (n)				
Proliferative	19	15	34	37
Secretory	28	16	47	48
Menstruation	8	9	-	-
Unknown	6	31	6	21
Contraception use (n)				
Yes	28	35	22	36
No	33	36	65	70
Fertility				
Yes	12	26	24	39
No	8	19	14	30
Unknown	41	26	49	37
Pain (n)				
Dysmenorrhea				
Yes	48	44	70	83
No	13	25	17	22
Unknown	-	2	-	1
Dyspareunia				
Yes	26	39	40	62
No	32	30	41	44
Unknown	3	2	6	-
Dyschezia				
Yes	18	26	27	44
No	43	45	56	62
Unknown	-	-	4	-
Dysuria				
Yes	11	15	19	29
No	50	56	68	77
Unknown	-	-	-	-

BMI is given as median ± SD (standard deviation); n, number of samples; EN^−^, without endometriosis; EN^+^, with endometriosis; BMI, body mass index; unknown, data not available; and pain, where yes denotes mild to strong pain on a nominal rating scale (NRS) of 2–10.

**Table 3:** Quantification of MT1-MMP staining in eutopic endometrium, adenomyosis, and ovarian endometriosis using HSCORE and percentage of stained glands.

	EM ^a^	OV ^b^	Adenomyosis ^c^
	HSCORE		
Mean	91	31	149
SEM	15.9	10.2	15.5
*p*-values	0.0325 ^a,b^	0.0003 ^b,c^	0.024 ^a,c^
N	16	6	20
Age	45 ± 6.6	38 ± 8.4	44.5 ± 5.0
	Percentage of stained glands		
Mean	65	32	92
SEM	9.2	3.0	10.5
*p*-values	n.s. ^a,b^	0.0002 ^b,c^	0.037 ^a,c^
N	16	6	20
Age	45 ± 6.6	38 ± 8.4	44.5 ± 5.0

Age is given as median ± SD (standard deviation). SEM, standard error of the mean; EM, endometrium; AD, adenomyosis; OV, ovarian endometriosis; n.s., not significant; N, number of samples. n.s. ^a,b^ means that group a is not significantly different from group b. ^b,c^ means that b is significantly different from c. ^a,c^ means that a is significantly different from c. Analysis was performed using the Mann–Whitney test.

**Table 4:** MT1-MMP levels in serum samples in the cycle phases.

	Without Endometriosis			With Endometriosis		
Phases	Menstrual ^a^	Proliferative ^b^	Secretory ^c^	Menstrual ^d^	Proliferative ^e^	Secretory ^f^
Samples (n)	8	18	29	9	15	16
Median age	26.5 ± 10.4	27 ± 8.7	28 ± 7.6	31 ± 6.3	37 ± 6.3	32 ± 6.6
Mean (ng/mL)	0.7	0.6	0.8	0.7	4.1	0.6
SEM	0.3	0.2	0.3	0.3	3.7	0.4
Range (ng/mL)	0.1–2.9	0–3.3	0–7.7	0–2.2	0–57.2	0–5.7
*p*-values		n.s. ^a,b^	n.s. ^a,c^ n.s. ^b,c^	n.s. ^a,d^	≤0.05 ^d,e^ 0.042 ^b,e^	≤0.05 ^e,f^ n.s. ^d,f^ n.s. ^c,f^

Age is given as median ± SD (standard deviation). SEM, standard error of the mean; n, number of samples. n.s. ^a,b^, n.s. ^a,c^, n.s. ^b,c^, n.s. ^a,d^, n.s. ^d,f^, n.s. ^c,f^ mean that a is not significantly different from b, a from c, b from c, a from d, d from f and c from f, respectively. ≤0.05 ^d,e^ indicates that d is significantly different from e; 0.042 ^b,e^ indicates that b is significantly different from e; ≤0.05 ^e,f^ indicates that e is significantly different from f. The Mann-Whitney test was used.

**Table 5:** Concentrations of MT1-MMP in serum samples of patients with and without endometriosis (A) and with and without contraception (B).

A	Without Endometriosis		With Endometriosis	
Samples (n)	61		71	
Median age	27 ± 7.9		34 ± 7.1	
Mean (ng/mL)	0.7		1.3	
SEM	0.2		0.8	
Range (ng/mL)	0–7.7		0–57.2	
*p*-value			0.0016	
**B**	**Contraception**			
	**w/o EN, w/o c ^a^**	**w/o EN, w c ^b^**	**w EN, w/o c ^c^**	**w EN, w c ^d^**
Samples (n)	33	28	36	35
Median age	28 ± 9.2	25 ± 4.9	34 ± 7.3	34 ± 6.9
Mean (ng/mL)	0.8	0.5	0.7	2.0
SEM	0.3	0.2	0.2	1.6
Range (ng/mL)	0–7.7	0–3.3	0–5.7	0–57.2
*p*-values		n.s. ^a,b^	n.s. ^b,c^ 0.043 ^a,c^	n.s. ^c,d^ 0.030 ^b,d^

(A,B) Age is given as median ± SD (standard deviation). SEM, standard error of the mean; (B) w/o EN, w/o c, without endometriosis and without contraception; w/o EN, w c, without endometriosis using contraception; w EN, w/o c, with endometriosis and without contraception; w EN, w c, with endometriosis using contraception; n, number os samples; n.s., not significant. n.s. ^a,b^, n.s. ^b,c^, n.s. ^c,d^ indicates that a and b are not significantly different, b not from c, and c not from d, respectively; 0.043 ^a,c^ indicates that a is significantly different from c; indicates that c and d are not significantly different; 0.030 ^b,d^ indicates that d is significantly different from f. The Kruskall-Wallis (A) and Mann–Whitney test (B) were used for analysis.

**Table 6:** MT1-MMP levels in endocervical mucus samples during the cycle phases.

	Without Endometriosis		With Endometriosis	
	Proliferative ^a^	Secretory ^b^	Proliferative ^c^	Secretory ^d^
Samples (n)	32	49	34	51
Median age	31 ± 9.1	27 ± 7.4	30.5 ± 6.4	34 ± 6.5
Mean (ng/mL)	4.1	2.8	4.6	3.5
SEM	0.3	0.2	0.4	0.3
Range (ng/mL)	0–7.6	0–6.9	0–14	0–9.3
*p*-values		0.0006 ^a,b^	n.s. ^b,c^ n.s. ^a,c^	0.01 ^c,d^ n.s. ^b,d^

Age is given as median ± SD (standard deviation). SEM, standard error of the mean; n.s., not significant; n, number of samples; 0.0006 ^a,b^ indicates that a and b are significantly different; 0.01 ^c,d^ indicates that c and d are significantly different; n.s. ^b,c^, n.s. ^a,c^, n.s.^b,d^ indicates that b is not significant different from c, a not from c, and b not from d, respectively. The Mann–Whitney test was used for analysis.

**Table 7:** MT1-MMP levels in endocervical mucus samples of patients with and without endometriosis and with or without contraception.

A	Without Endometriosis		With Endometriosis	
Samples (n)	87		106	
Median age	28 ± 8.2		33 ± 7.1	
Mean (ng/mL)	3.2		3.6	
SEM	0.2		0.2	
Range (ng/mL)	0–7.6		0–14	
*p*-value			n.s.	
**B**	**Contraception**			
	**w/o EN, w/o ^c^ a**	**w/o EN, w c ^b^**	**w EN, w/o c ^c^**	**w EN, w c ^d^**
Samples (n)	65	22	70	36
Median age	30 ± 8.5	27 ± 7.2	32.5 ± 7.2	33 ± 6.8
Mean (ng/mL)	3.4	2.7	3.8	3.2
Range (ng/mL)	0–7.6	0–5.4	0–9.3	0–14
SEM	0.2	0.4	0.2	0.4
*p*-values		n.s. ^a,b^	n.s. ^a,c^ n.s. ^b,c^	0.003 ^c,d^ n.s. ^b,d^

Age is given as median ± SD (standard deviation). SEM, standard error of the mean; w/o EN, w/o c, without endometriosis and without contraception; w/o EN, w c, without endometriosis using contraception; w EN, w/o c, with endometriosis and without contraception; w EN, w c, with endometriosis using contraception; n, number of samples; n.s., not significant; n.s. ^a,b^, n.s. ^a,c^, n.s. ^b,c^, and n.s. ^b,d^ indicate that a is not significantly different from b, a not from c, b not from c, and b not from d, respectively; 0.003 ^c,d^ indicates that c is significantly different from d. The Mann–Whitney test was used for statistical analysis.

**Table 8:** Relationship of MT1-MMP levels in serum (A) and endocervical mucus samples (B) with different clinical characteristics.

A	Serum MT1-MMP Levels					
				Mean (ng/mL) ± SEM		
	W/o EN	W EN	*p*-Values	Without Pain	With Pain	*p*-Values
BMI (kg/m^2^)	23.9 ± 0.8	24.4 ± 0.6	n.s.			
Age	28.9 ± 1.0	34.8 ± 0.8	0.0001			
Dysmenorrhea	n = 130			0.97 ± 0.3	1.10 ± 0.7	n.s.
Dysuria	n = 131			1.18 ± 0.5	0.22 ± 0.1	n.s.
Dyschezia	n = 132			1.24 ± 0.6	0.52 ± 0.2	n.s.
Dyspareunia	n = 127			0.77 ± 0.2	1.46 ± 1.1	n.s.
**B**	**Endocervical Mucus MT1-MMP Levels**					
				**Mean (ng/mL) ± SEM**		
	**W/o EN**	**W EN**	***p*-Values**	**Without Pain**	**With Pain**	***p*-Values**
BMI (kg/m^2^)	22.9 ± 0.5	23.4 ± 0.4	n.s.			
Age	30.8 ± 0.9	33.9 ± 0.7	n.s.			
Dysmenorrhea	n = 192			3.18 ± 0.3	3.49 ± 0.2	n.s.
Dysuria	n = 193			3.39 ± 0.2	3.52 ± 0.3	n.s.
Dyschezia	n = 189			3.46 ± 0.2	3.50 ± 0.2	n.s.
Dyspareunia	n = 187			3.56 ± 0.2	3.39 ± 0.2	n.s.

(A) Correlation analysis of serum samples with clinical parameters. (B) Correlation analysis of endocervical mucus samples with clinical parameters. n, number of samples; n.s., not significant; SEM, standard error of the mean; BMI, body mass index; W/o EN, without endometriosis; W EN, with endometriosis. Cycle days (1–32); without pain (pain scale = 0–3); with pain (pain scale 4–10). BMI and age are given as mean ± SEM. The Mann–Whitney test was used.

## Data Availability

The data is available from the corresponding author upon request.
